# Genomic characteristics of carbapenem-non-susceptible *Cronobacter sakazakii* isolated from an ICU patient at the Songkhla Hospital, Thailand

**DOI:** 10.1128/mra.01281-23

**Published:** 2024-07-31

**Authors:** Siriporn Lalakorn, Kamonnut Singkhamanan, Arnon Chukamnerd, Sanicha Chumtong, Thitaporn Dechathai, Jirasa Boonsan, Siriwan Kompramool, Thanchanok Muangkaew, Thunchanok Yaikhan, Nutwadee Chintakovid, Sarunyou Chusri, Rattanaruji Pomwised, Monwadee Wonglapsuwan, Komwit Surachat

**Affiliations:** 1 Division of Biological Science, Faculty of Medicine, Prince of Songkla University, Songkhla, Thailand; 2 Department of Biomedical Sciences and Biomedical Engineering, Faculty of Medicine, Prince of Songkla University, Songkhla, Thailand; 3 Division of Infectious Diseases, Department of Internal Medicine, Faculty of Medicine, Prince of Songkla University, Songkhla, Thailand; 4 Translational Medicine Research Center, Faculty of Medicine, Prince of Songkla University, Songkhla, Thailand; University of Maryland School of Medicine, Baltimore, Maryland, USA

**Keywords:** carbapenem-non-susceptible *Cronobacter sakazakii*, whole-genome sequence

## Abstract

*Cronobacter sakazakii* is a pathogen that causes severe diseases such as meningitis and necrotizing enterocolitis in infants, associated with the consumption of rehydrated powder infant formula. We report a whole-genome sequence of carbapenem-non-susceptible *C. sakazakii* isolated from the nasopharynx of the patient admitted to the ICU ward, Songkhla Hospital, Thailand.

## ANNOUNCEMENT

*Cronobacter*, a gram-negative, facultatively anaerobic, oxidase-negative, catalase-positive genus within the Enterobacteriaceae family, includes desiccation-resistant species found in products, particularly powder infant formula (1). This bacterium is associated with severe diseases, such as meningitis, septicemia, and necrotizing enterocolitis (2, 3). However, the *Cronobacter* genome has not been well characterized. We aimed to sequence the entire genome of carbapenem-non-susceptible *C. sakazakii* and reported its genomic features.

*C. sakazakii* SK012 was isolated by nasopharyngeal swab of a 77-year-old hypertension patient from ICU in the Songkhla Hospital, Thailand on 21 May 2019. The patient was treated with ceftriaxone, ciprofloxacin, gentamicin, piperacillin/tazobactam, meropenem, and ertapenem. Access to the clinical data was approved by the Human Research Ethics Committee of Prince of Songkla University. The isolate was cultured on MacConkey agar with 2 µg meropenem at 37°C overnight. MALDI-TOF MS was used for the identification of bacterial species. For DNA extraction, a single colony was inoculated into 3 mL Tryptic Soy Broth and incubated at 37°C overnight. Genomic DNA was extracted using the GF-1 Bacterial DNA Extraction Kit, following the manufacturer’s instructions. The DNA library was constructed using the MGIEasy Library Prep Kit V1.1 (MGI Tech). Then, sequencing was conducted on an MGISEQ-2000 platform (BGI, Beijing, China), generating paired-end reads of 150 bp in length. A total of 4,070,902 paired-end reads generated by the sequencer underwent quality verification using FastQC v0.11.9 (4). BacSeq v1.0 (5), which integrates SPAdes v3.15.4 (6), Prokka v1.12 (7), QUAST v4.0 (8), and BUSCO v5.2.2 (9), facilitated the assembly and annotation of the reads. *In silico* species identification employed BLASTN against the NCBI database (10), and PubMLST database identified the sequence type (ST) as ST1, belonging to clonal complex 1 (11). Antimicrobial resistance genes (ARGs) were detected using the CARD database, with a ≥80% identity match (12). Default parameters were employed for all software, unless specified otherwise.

WGS of *C. sakazakii* SK012 generated 4,515,439 bp, with N50 value of 470,914 bp, 37 contigs, and 56.69% GC content. Prokka reported the amounts of 92 tRNA, 7 rRNA, 1 tmRNA, and 15 CRISPR arrays. Importantly, the strain carried many ARGs conferring resistance to several antimicrobial classes such as fluoroquinolone, cephalosporin, tetracycline, carbapenem, and aminoglycoside (Table 1). These ARGs are commonly encoded for antibiotic efflux pumps, antibiotic target alteration, and/or antibiotic inactivation. For carbapenem non-susceptibility, this strain harbored *marA* encoding for efflux pump, which might be associated with multiple antibiotic-resistant phenotypes, especially carbapenem (13).

**TABLE 1:** ARGs identified in *C. sakazakii* SK012^*a*^

ARGs	SNPs	Antibiotics
*CRP*		Macrolide, fluoroquinolone, penam
*CSA-1*		Cephalosporin
Mutation in EF-Tu	R234F	Elfamycin
Mutation in GlpT	E448K	Phosphonic acid
*msbA*		Nitroimidazole
*emrB*		Fluoroquinolone
H-NS		Macrolide, fluoroquinolone, cephalosporin, cephamycin, penam, tetracycline
*emrR*		Fluoroquinolone
*marA*		Fluoroquinolone, monobactam, carbapenem, cephalosporin, glycylcycline, cephamycin, penam, tetracycline, rifamycin, phenicol
Mutation in MarR	S3N	Fluoroquinolone, cephalosporin, glycylcycline, penam, tetracycline, rifamycin, phenicol
*rsmA*		Fluoroquinolone, diaminopyrimidine, phenicol
*acrA*		Fluoroquinolone, cephalosporin, glycylcycline, penam, tetracycline, rifamycin, phenicol
*kpnF*		Macrolide, aminoglycoside, cephalosporin, tetracycline, rifamycin
*kpnE*		Macrolide, aminoglycoside, cephalosporin, tetracycline, rifamycin
*FosA8*		Phosphonic acid
Mutation in PBP3	D350N, S357N	Cephalosporin, cephamycin, penam
*adeF*		Fluoroquinolone, tetracycline
*vanG*		Glycopeptide

^*a*^ SNP, single-nucleotide polymorphism.

## Data Availability

The genome sequence has been deposited in GenBank database under BioProject accession number PRJNA1049972, BioSample accession number SAMN38721045, SRA accession number SRR27308067, and genome accession number JAXOAS000000000.
